# pH-Responsive ZIF-8 Precisely Induces Apoptosis of Oral Squamous Cell Carcinoma over Orofacial Mesenchymal Stem Cells

**DOI:** 10.3390/pharmaceutics18030394

**Published:** 2026-03-22

**Authors:** Jessica Hao, Mehrnaz Zakershahrak, Peter Ly, Xiaobin Huang, Kunfeng Sun, Shilan Zhang, Fusun Ozer, Chider Chen

**Affiliations:** 1 Department of Oral & Maxillofacial Surgery & Pharmacology, School of Dental Medicine, University of Pennsylvania, Philadelphia, PA 19104, USA; haoje@dental.upenn.edu (J.H.); mehrnazz@upenn.edu (M.Z.); bmwzzhsh@upenn.edu (X.H.); kfsun@dental.upenn.edu (K.S.); shilanz@upenn.edu (S.Z.); 2 Department of Biology, College of Arts and Sciences, University of Pennsylvania, Philadelphia, PA 19104, USA; peterly@sas.upenn.edu; 3 Department of Preventive and Restorative Sciences, School of Dental Medicine, University of Pennsylvania, Philadelphia, PA 19104, USA; ozerf@upenn.edu; 4 Center of Innovation & Precision Dentistry, School of Dental Medicine, School of Engineering and Applied Sciences, University of Pennsylvania, Philadelphia, PA 19104, USA

**Keywords:** oral squamous cell carcinoma (OSCC), drug delivery, mesenchymal stem/stromal cells (MSCs), zeolite imidazolate framework-8 (ZIF-8), apoptosis

## Abstract

**Objectives**: pH-responsive zeolite imidazolate framework-8 (ZIF-8) enables selective release of 5-fluorouracil (5-FU) within the acidic tumor microenvironment. However, the direct effects of ZIF-8 itself on cancer cells or surrounding tissues remain unclear. Since oral cancer involves interactions between epithelial tumor cells and stromal cells, comparing the effects of ZIF-8 on epithelial cancer cells and orofacial mesenchymal stem/stromal cells (OMSCs) is critical to understanding its broader biological impact. **Methods**: The effects of ZIF-8 on SCC7 epithelial cancer cells and OMSCs, including periodontal ligament stem cells (PDLSCs) and dental pulp stem cells (DPSCs), were evaluated using RNA sequencing, nuclear staining, live/dead assays, and immunocytochemistry. Cells were treated with 0, 1, 10, or 100 μg/mL ZIF-8. Based on nuclear staining results, live/dead viability assays were conducted on SCC7 and DPSCs treated with 0 or 100 μg/mL ZIF-8. Apoptosis-related markers (Bax, caspase-3, caspase-6, and caspase-10) were assessed following exposure to 100 μg/mL ZIF-8. **Results**: Transcriptomic analysis revealed that ZIF-8 not only facilitates selective 5-FU release but also directly induces apoptosis in SCC7 cells compared with 5-FU alone. At 100 μg/mL ZIF-8, SCC7 viability was significantly reduced, whereas OMSC viability was preserved. Nonviable SCC7 cells increased markedly compared with controls, while DPSCs showed no significant change. Apoptosis-related signaling was also elevated in SCC7 cells compared with DPSCs. **Conclusions**: ZIF-8 at 100 μg/mL selectively inhibits SCC7 growth while sparing OMSC viability and apoptosis.

## 1. Introduction

Nearly 90% of oral and oropharyngeal cancers are classified as head and neck squamous cell carcinoma (HNSCC), with global estimates exceeding 350,000 new diagnoses and 170,000 deaths each year [1] and a five-year mortality rate of nearly 50% [2]. In individuals with advanced-stage tumors, there is a significant likelihood of invasion into nearby tissues, accompanied by lymph node involvement, distant metastases, and an unusually elevated risk of developing a second malignancy over the course of their lifetime [3]. The inability of current diagnostic methods to consistently distinguish between normal and tumor tissue can delay treatment [4]. Head and neck radiation therapy results in a range of side effects for cancer patients, including both acute and chronic soft tissue alterations and temporary or permanent sensory disturbances [5]. Resistance to chemotherapeutic and biological agents undermines the efficacy of numerous current therapeutic approaches [6]. Thus, effective oral cancer treatment relies on multifaceted approaches that optimize tumor control while minimizing treatment-related adverse effects [7]. Given the challenges associated with oral cancer treatment, there is an urgent need for new and effective therapeutic approaches.

Oral cancer not only resides in the transformed epithelium but also interacts with the surrounding stroma [8]. Orofacial mesenchymal stem/stromal cells (OMSCs) are overabundant in oral squamous cell carcinoma (OSCC), facilitating tumor growth by impacting stromal development and hindering the systemic immune response [9]. These stromal populations can also influence their environment by secreting biomolecules, facilitating cell–cell interactions, inhibiting immune cell protective functions, promoting angiogenesis, or differentiating into other tumor stroma components, such as cancer-activated fibroblasts (CAF) [10]. They can prevent the apoptosis of cancer stem cells (CSCs) and stimulate their proliferation, thereby contributing to cancer progression, drug resistance, metastasis, and recurrence [11]. In contrast, MSCs show antitumor effects through increasing G1 phase cell cycle arrest of tumor cells [12], inhibiting tumor cell migration and invasion [13], suppressing tumor vascularization [14], reducing inflammatory infiltration, and regulating oncogenes [15]. MSCs can selectively target and accumulate at tumor sites, thereby offering therapeutic advantages by enhancing treatment efficacy and minimizing systemic side effects [15]. Their dual roles in cancer cells and the mechanisms driving their functional switch remain poorly understood [11,16]. Observing the different effects of therapeutics on cancer and stem cells is crucial to understanding their holistic impact on the body. The porous zeolite imidazolate framework-8 (ZIF-8), composed of Zn2+ and 2-methylimidazole, has attracted considerable research attention owing to its simple preparation, exceptionally high surface area, and remarkable thermal stability [17]. While stable under physiological conditions (pH 7.4), ZIF-8 is susceptible to degradation in acidic environments [18], making it an ideal platform for pH-responsive drug delivery. Particularly in the tumor microenvironment, increased metabolic activity through the Warburg Effect [19] and inadequate perfusion lead to acidification (pH 5.5) due to accumulation of metabolic waste products [20]. ZIF-based carriers, when loaded with anticancer drugs, offer a potential alternative strategy to improve therapeutic efficacy while overcoming limitations of conventional treatments [21]. Our previous experimental studies also demonstrated that commercially available ZIF-8 can absorb and release 5-fluorouracil (5-FU) in a controlled, pH-responsive manner. Through this mechanism, ZIF-8 and 5-FU act synergistically to suppress the proliferation of OSCC cells [22].

Examining the effects of ZIF-8 on both OMSC and OSCC cells is essential for understanding its overall impact on oral cancer. Although ZIF-8 has emerged as a promising nanocarrier for chemotherapeutic delivery, its direct influence on stromal and tumor compartments is not well defined. Since the tumor microenvironment involves dynamic interactions between stromal and malignant cells, assessing differential responses is critical for predicting therapeutic outcomes. Investigating whether ZIF-8 exerts differential cytotoxicity, protective, or modulatory effects on OMSCs compared to OSCC cells will not only clarify its potential as a drug delivery system but also provide insights into how such nanostructures may alter stromal–tumor crosstalk. This study, therefore, aims to characterize the in vitro effects of ZIF-8 on SCC compared to OMSCs, providing insight into its potential role in modulating stromal–tumor interactions and informing future translational applications in oral cancer.

## 2. Materials and Methods

### 2.1. Materials

ZIF-8 (C_8_H_10_N_4_Zn, ≥99% purity, molecular weight: 227.58) with a particle size of 4.9 μm and surface area of 1300–1800 m^2^/g was obtained from Sigma-Aldrich (St. Louis, MO, USA). Prior to use, the ZIF-8 was heat-activated at 160 °C for 24 h in a laboratory oven (Binder ED056) supplied by LabRepCo. (Horsham, PA, USA). The LIVE/DEAD Viability/Cytotoxicity kit was acquired from Invitrogen. The immunofluorescence images were captured through an Olympus IX71 Microscope System. The SCC7 (RRID: CVCL_V412) cell line was kindly provided by Dr. Anh Le at the University of Pennsylvania School of Dental Medicine.

DMEM/RPMI-1640, 200 mM glutamate, penicillin-streptomycin, L-ascorbic acid phosphate, and TrypLE Express were purchased from Thermo Fisher Scientific. Fetal Bovine Serum was purchased from Hyclone Laboratories.

### 2.2. Cell Culturing

SCC7 (oral squamous cell carcinoma cell line) was cultured in alpha MEM supplemented with 10% (*v*/*v*) FBS, 1% (*v*/*v*) 200 mM glutamate, and 1% (*v*/*v*) penicillin/streptomycin. Periodontal ligament stem cells (PDLSC) and Dental pulp stem cells (DPSC) were used as representatives of the stromal population in the OSCC microenvironment. PDLSC and DPSC were cultured in alpha MEM supplemented with 15% (*v*/*v*) FBS, 1% (*v*/*v*) 200 mM glutamate, 1% (*v*/*v*) L-ascorbic acid phosphate, and 1% (*v*/*v*) penicillin/streptomycin. Cryopreserved cells were rapidly thawed in a 37 °C water bath for approximately 1 min. The cells were resuspended in 1 mL of prepared culture medium and centrifuged at 1300 rpm for 5 min. After centrifugation, the supernatant was carefully removed, and the cell pellet was resuspended in 1 mL of fresh medium. The cell suspension was then transferred to a culture dish, followed by the addition of 9 mL of complete medium. Cultures were maintained at 37 °C until they reached full confluence. The culture medium was refreshed every two days with 10 mL of newly prepared medium.

After confluence, the cells were passed onto two additional dishes for continued proliferation throughout the experiment. After removing the medium, 3 mL of TrypLE Express was added to detach cells. The cells were incubated for 5 min at 37 °C and gently agitated until they were visibly detached. To neutralize the TrypLE Express, 3 mL of fresh medium was added, and the culture was centrifuged at 1300 rpm for 5 min. The pellet was resuspended in 1 mL of fresh medium. A portion of the resuspended cells was added to each new dish containing 10 mL of fresh medium. The dishes were then incubated at 37 °C in a 5% CO_2_ atmosphere until the cells reached 100% confluence. This procedure was repeated to maintain the cells throughout the experiments.

### 2.3. RNA Sequencing Analysis

SCC7 cells were passaged into 6-well culture plates and incubated at 37 °C in a 5% CO_2_ atmosphere until the cells reached 100% confluence. 5-FU and 5-FU-loaded ZIF-8 at 50 μg/mL [22] were added to the culture medium for 72 h. After treatment, cells were collected for total RNA isolation using the QIAzol Lysis Reagent protocol with the RNeasy Kit (Qiagen, Hilden, Germany). The cDNA library was synthesized and sequenced by Novogene (Beijing, China) using an Illumina sequencer. Reads obtained from RNA-seq were then aligned to the reference genome. The differential expression between conditions was statistically assessed, and genes with FC > 1 or FC < −1 and *p* value < 0.01 were identified as differentially expressed. GO functional classification of differentially expressed genes was defined based on the QuickGO database. Gene set enrichment analysis (GSEA) was also performed by using the GSEA software (MSigDB v2023.2).

### 2.4. Nuclear Staining

The in vitro cytotoxicity of ZIF-8 was assayed against SCC7, PDLSC, and DPSC, and cell viability was visualized via toluidine blue nuclear staining. Cells were seeded into 12-well culture plates (*n* = 3) and allowed to adhere overnight at 37 °C in a humidified atmosphere containing 5% CO_2_. ZIF-8 was added to the culture medium at 0 (control), 1, 10, and 100 μg/mL. After removing the medium at 72 h, a toluidine blue stain was performed. After 8 h of incubation, the staining solution was removed, the wells were washed with PBS, and the wells were air-dried. Cell viability of SCC7 was compared with that of PDLSC and DPSC at various concentrations of ZIF-8 relative to the control. An optimal concentration that most clearly distinguishes between the effects on the cell types was chosen for subsequent experiments.

To quantify the nuclear staining results, the open-source software FIJI (ImageJ2) was used. Plate images were opened in the software and converted to grayscale (8-bit) to assess overall intensity. For color-specific analysis, images were split into red, green, and blue (RGB) channels. Since toluidine blue strongly absorbs light in the red–green portion of the spectrum while transmitting blue light, the blue channel provided the strongest signal for quantification and was selected. Regions of interest (ROIs) were drawn around each well using the oval selection tool, and all ROIs were added to the ROI Manager. Mean gray values within each ROI were measured using the “Measure” function. In 8-bit grayscale, pixel intensity values range from 0 (black, representing deepest staining) to 255 (white, no staining); thus, lower mean gray values indicate greater staining intensity. An unpaired Student’s *t*-test was conducted using GraphPad Prism version 10 (GraphPad Software, La Jolla, CA, USA). A *p*-value < 0.05 was considered statistically significant.

### 2.5. LIVE/DEAD Staining

Based on these results, an optimal concentration (100 μg/mL) of ZIF-8 was compared with the control using the LIVE/DEAD Viability/Cytotoxicity kit (Invitrogen, Waltham, USA). The cultured SCC7, PDLSC, and DPSC were passed to 12-well tissue culture plates (*n* = 3) containing round glass coverslips and incubated overnight at 37 °C and 5% CO_2_. ZIF-8 was added to the culture medium at 0 μg/mL (control) and at the optimal concentration determined in the nuclear staining experiment. After 72 h, the culture medium was removed and the coverslips extracted. A working solution of 4 μM Ethidium Homodimer-1 (EthD-1) and 2 μM calcein AM was mixed and added to the coverslips. After mounting the coverslips, the number of viable (green) and nonviable (red) cells was counted using a fluorescence microscope. The proportion of nonviable/viable cells was calculated and compared between the sample groups.

### 2.6. Immunocytochemistry

Finally, SCC7, DPSC, and PDLSC were exposed to ZIF-8 at 100 μg/mL and compared for the presence of apoptotic proteins BAX, Caspase-3 (CAS3), Caspase-6 (CAS6), and Caspase-10 (CAS10). The cultured cells were passed to 12-well tissue culture plates (*n* = 3) containing round glass coverslips and incubated overnight at 37 °C and 5% CO_2_. ZIF-8 was added to the culture medium at 0 μg/mL (control) and 100 μg/mL. After 72 h, the cells were fixed with 4% PFA for 10 min. Next, the coverslips were extracted from the wells, permeabilized with 0.1% TritonX for 30 min, and blocked with 3% goat serum for 15 min. The primary antibodies were added at a 1:200 dilution (1% BSA, 0.1 M Glycine, and 0.1% NaN_3_/PBS) and incubated overnight at 4 °C. The primary antibody solution contained BMI-1 (D42B3/#5856; Cell Signaling Technology, Danvers, MA, USA) for OSCC and OCT4 (AB3209; Millipore, Billerica, MA, USA) for OMSC, as well as antibodies against apoptotic proteins Bax (MA5-14003/6A7; Thermo Fisher Scientific, Waltham, MA, USA), Caspase-3 (sc-7272; Santa Cruz Biotechnology, Dallas, TX, USA), Caspase-6 (sc-1231; Santa Cruz Biotechnology, Dallas, TX, USA), and Caspase-10 (sc-393983; Santa Cruz Biotechnology, Dallas, TX, USA). The corresponding secondary antibodies were then added at a 1:200 dilution (1% BSA, 0.1 M Glycine, and 0.1% NaN_3_/PBS) and incubated for 30 min, protected from light. At last, the coverslips were mounted onto microscope slides with DAPI to highlight cell nuclei. Under a fluorescence microscope, blue immunofluorescence illuminated the cell nuclei, red immunofluorescence demonstrated specificity for the type of cell, and green immunofluorescence highlighted cells undergoing apoptosis that expressed proteins associated with apoptosis. The proportions of the colors were calculated and compared across the sample groups.

## 3. Results

### 3.1. Transcriptomic Analysis Showed ZIF-8 Selectively Induces SCC7 but Not OMSC Apoptosis

**Figure 1:**
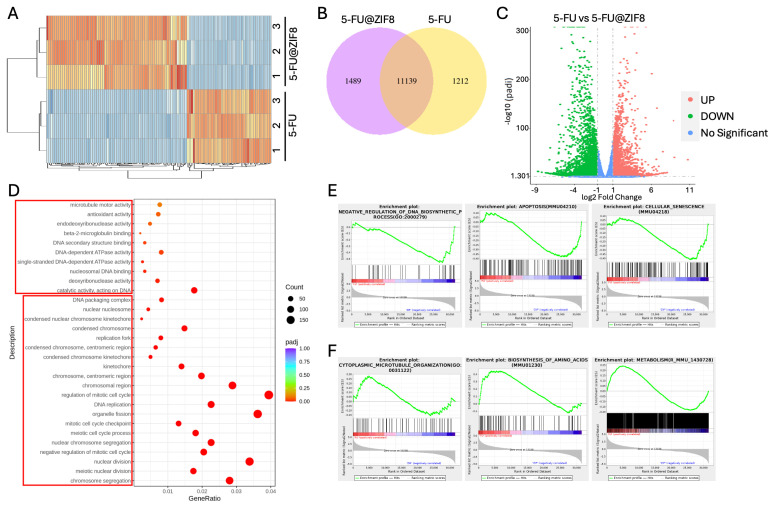
Transcriptome analysis showed that ZIF treatment significantly promotes cell apoptosis and cellular senescence in SCC7 cells. (**A**) Heat map of RNA-seq expression data showing the genes that were differentially regulated following treatment with either 5-FU or 5-FU with ZIF-8. Differentially expressed genes were selected based on a fourfold change. (**B**) We found 2701 transcripts that significantly change their expression, with log2 fold change (FC) > 1 or FC < −1 and *p* value < 0.05, between 5-FU and 5-FU with ZIF-8 treatments. (**C**) Volcano plot with the log2 fold changes in gene expression on the x-axis and the statistical significance (*p*-value) on the y-axis between 5-FU and 5-FU with ZIF-8 treatments. (**D**) Enrichment analysis of Gene Ontology (GO) terms over the 2701 ZIF-8 target genes revealed that the most enriched terms were associated with DNA structure, cell cycle, and cellular apoptosis. (**E**,**F**) Gene set enrichment analysis (GSEA) further confirmed enrichment of cell cycle-related pathways, DNA and protein biosynthesis, and apoptosis in SCC7 cells following 5-FU treatment with ZIF-8.

Our previous study demonstrated that pH-responsive ZIF-8 selectively releases 5-FU into the acidic tumor microenvironment, playing a synergistic role in inhibiting the proliferation of SCC7 epithelial oral cancer cells [22]. This finding prompted us to map the transcriptomic profiles between 5-FU and 5-FU-loaded ZIF-8 treatment in SCC7 cells. We found 2701 transcripts that significantly change their expression, log2 fold change (FC) > 1 and FC < −1 and *p* value < 0.01, after 5-FU-loaded ZIF-8 treatment compared to the 5-FU alone group (Figure 1A). Among these genes, 1489 (55.13%) were up-regulated and 1212 (44.87%) were down-regulated upon 5-FU-loaded ZIF-8 treatment (Figure 1B,C). Enrichment analysis of gene ontology (GO) terms over the 2701 ZIF-8 target genes showed that the most enriched were associated with DNA structure, cell cycle, and cellular apoptosis (Figure 1D). These data were further confirmed by Gene Set Enrichment Analysis (GSEA) to determine the downregulation of cell cycle-related pathways, DNA and protein biosynthesis, and metabolism, and upregulation of apoptosis and cellular senescence in SCC7 cells following 5-FU-loaded ZIF-8 treatment compared to the 5-FU alone group (Figure 1E). All these findings indicated that ZIF-8 might directly induce apoptosis of epithelial SCC cancer cells.

### 3.2. Cell Viability Assays Confirmed That ZIF-8 Selectively Induces SCC7 Apoptosis

**Figure 2:**
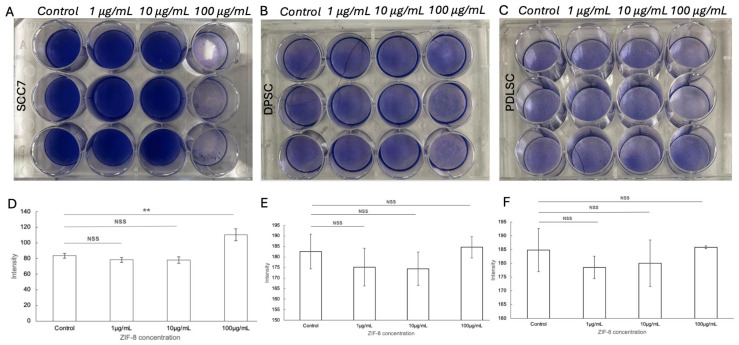
ZIF8 treatment significantly increased apoptosis in SCC7 cells but not in orofacial MSCs. Toluidine blue staining of SCC7 (**A**), DPSC (**B**), and PDLSC (**C**) when treated with various concentrations of ZIF-8. The results of the statistical analysis are depicted in (**D**–**F**), respectively. ** *p* < 0.01. Error bars: x ± SD. NSS: Not statistically significant.

To address this question, we next aimed to investigate whether ZIF-8 treatment alone could regulate SCC7 cell viability. Since oral cancer interacts with the abundant surrounding stroma [8], we then used OMSCs as the control group to identify the roles of ZIF-8 in OSCC therapy. Nuclear staining demonstrated statistically significantly lower SCC7 viability compared to OMSCs at 100 μg/mL ZIF-8 treatment (Figure 2A–C). Lower concentrations of ZIF-8, at 1 μg/mL and 10 μg/mL, showed similar viability of SCC7 compared to the control (Figure 2A). PDLSC and DPSC exhibited similar cell viability at all concentrations compared to the control group (Figure 2A,B). Due to the distinctive difference in cell viability between SCC7 and OMSCs at 100 μg/mL ZIF-8 treatment, this concentration was selected as the optimum concentration for subsequent experiments.

**Figure 3:**
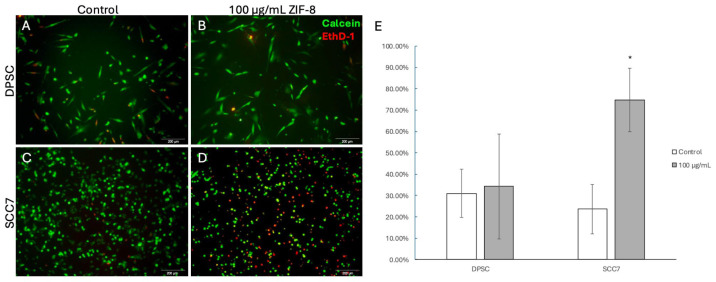
Cell viability analysis showed increased cytotoxicity in SCC7 cells following ZIF-8 treatment. (**A**) DPSCs with 0 μg/mL ZIF-8 treatment. (**B**) DPSCs with 100 μg/mL ZIF-8 treatment. (**C**) SCC7 with 0 μg/mL ZIF-8 treatment. (**D**) SCC7 with 100 μg/mL ZIF-8 treatment. The proportion of red fluorescence (EthD-1 positive staining) was significantly higher in SCC7 than in DPSCs at 100 μg/mL ZIF-8 treatment. (**E**) Statistical analysis depicting results. Scale bar, 200 μm. * *p* < 0.05. Error bars: x ± SD.

To further confirm whether ZIF-8 selectively induces SCC cell apoptosis in the tumor microenvironment, the LIVE/DEAD Viability/Cytotoxicity protocol was applied to SCC7 and DPSCs with or without ZIF-8 treatment. Calcein AM green fluorescence staining was used to identify non-toxic live cells, while EthD-1 red fluorescence was used to detect dead cells with nucleic acid staining. Our data indicated that the proportion of nonviable DPSCs remained similar between the 100 μg/mL ZIF-8 treatment and the control (Figure 3A,B); however, the proportion of nonviable SCC7 cells was significantly higher at the 100 μg/mL ZIF-8 treatment than in the control group (Figure 3C–E). These data indicate that ZIF-8 at 100 μg/mL has an inhibitory effect on epithelial SCC cancer cells but has no significant effect on the viability and apoptosis of OMSCs.

### 3.3. ZIF-8 Selectively Induces SCC7 Apoptosis Through Cell Death Pathways

**Figure 4:**
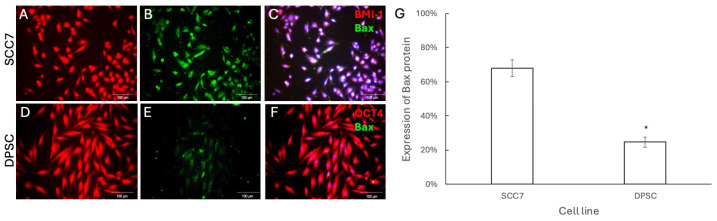
Immunofluorescent staining showed an increased BAX expression, a cell apoptosis marker, in SCC7 cells after treatment with ZIF-8. (**A**) SCC7 was stained with BMI-1 rabbit antibody, followed by anti-rabbit 2nd antibody with red immunofluorescence after treatment with ZIF-8. (**B**) An antibody against BAX (mouse) was used to detect apoptotic cells, followed by an anti-goat secondary antibody with green immunofluorescence for SCC cells treated with ZIF-8. (**C**) DAPI was used to highlight cell nuclei. (**D**) DPSCs were stained with OCT4 rabbit antibody, followed by anti-rat 2nd antibody with red immunofluorescence after treatment with ZIF-8. (**E**) An antibody against Bax was used to detect apoptotic DPSCs. (**F**) DAPI used to highlight cell nuclei. (**G**) Statistical analysis summarizing results across the sample groups. Scale bar, 100 μm. * *p* < 0.05. Error bars: x ± SD.

**Figure 5:**
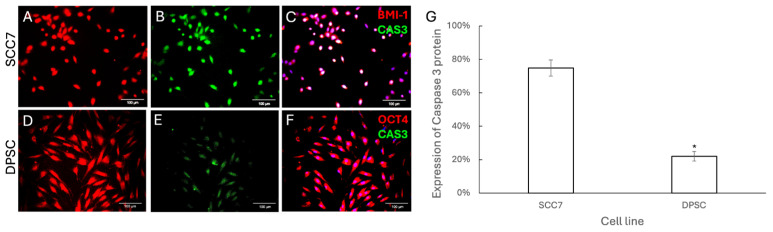
Immunofluorescent staining showed an increased level of Caspase-3, a central molecule in apoptotic pathways, in SCC7 cells after treatment with ZIF-8. (**A**) SCC7 was stained with BMI-1 for red immunofluorescence after ZIF-8 treatment. (**B**) An antibody against CAS3 (mouse) was used to detect activation of apoptotic pathways, followed by an anti-mouse secondary antibody with green immunofluorescence for SCC cells treated with ZIF-8. (**C**) DAPI was used to highlight cell nuclei. (**D**) DPSCs were stained with OCT4 for red immunofluorescence after treatment with ZIF-8. (**E**) An antibody against CAS3 was used to detect apoptotic DPSCs following ZIF-8 treatment. (**F**) DAPI was used to highlight cell nuclei. (**G**) Statistical analysis summarizing results across the sample groups. Scale bar, 100 μm. * *p* < 0.05. Error bars: x ± SD.

We then investigated whether ZIF-8 treatment significantly activates cell death signaling to induce apoptosis in epithelial cancer cells, but not in OMSCs. To achieve this goal, we first used BMI-1 as an SCC epithelial cell marker to label all SCC7 cells and used OCT4 as an OMSC marker to label all DPSCs. As BAX is a crucial player in programmed cell death [23], we first detected BAX levels to reveal whether ZIF-8 treatment could induce SCC7, but not OMSC, apoptosis. Immunochemistry staining revealed higher levels of BAX in SCC7 (Figure 4A–C) at 100 μg/mL ZIF-8 compared to DPSCs (Figure 4D–G). Next, we aimed to examine whether ZIF-8 treatment could activate the programmed death pathway to induce SCC7 apoptosis. As CAS-3 is a central molecule in apoptotic pathways [24], we then performed immunofluorescent staining to evaluate CAS-3 levels in SCC7 and DPSCs after ZIF-8 treatment. Our data indicated that ZIF-8 treatment indeed activated apoptotic signaling to induce SCC7 apoptosis, but not DPSC (Figure 5).

**Figure 6:**
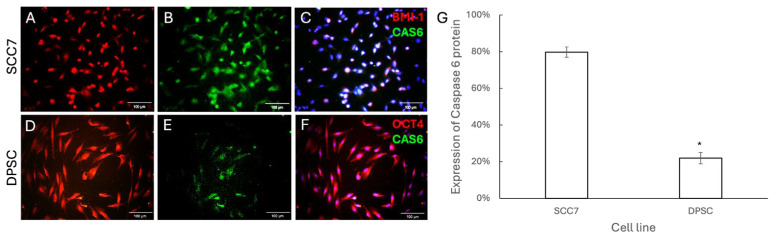
Caspase-6, a key regulator of pyroptosis, apoptosis, and necroptosis, was significantly elevated in SCC7 cells after treatment with ZIF-8. (**A**) SCC7 was stained with BMI-1 for red immunofluorescence after ZIF-8 treatment. (**B**) An antibody against CAS6 (goat) was used to detect caspase 6 levels, followed by an anti-goat secondary antibody with green immunofluorescence in SCC cells treated with ZIF-8. (**C**) DAPI was used to highlight cell nuclei. (**D**) DPSCs were stained with OCT4 for red immunofluorescence after treatment with ZIF-8. (**E**) An antibody against CAS6 was used to detect caspase-6 levels in DPSCs after ZIF-8 treatment. (**F**) DAPI was added to highlight cell nuclei. (**G**) Statistical analysis summarizing results across the sample groups. Scale bar, 100 μm. * *p* < 0.05. Error bars: x ± SD.

**Figure 7:**
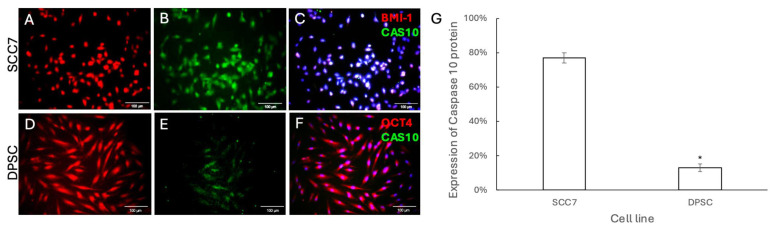
The death receptor-related apoptotic pathway was significantly increased in SCC7 cells after treatment with ZIF-8. (**A**) SCC7 was stained with BMI-1 for red immunofluorescence after ZIF-8 treatment. (**B**) An antibody against CAS10 (mouse) was used to detect the levels of caspase 10, followed by an anti-goat secondary antibody with green immunofluorescence for SCC cells treated with ZIF-8. (**C**) DAPI was used to highlight cell nuclei. (**D**) DPSCs were stained with OCT4 for red immunofluorescence after treatment with ZIF-8. (**E**) An antibody against CAS10 was used to detect caspase 10 levels in DPSCs after ZIF-8 treatment. (**F**) DAPI was used to highlight cell nuclei. (**G**) Statistical analysis summarizing results across the sample groups. Scale bar, 100 μm. * *p* < 0.05. Error bars: x ± SD.

Under certain conditions, three pathways of programmed cell death, including apoptosis, necroptosis, and pyroptosis, transform from one into another, with Caspase-6 playing a key role in regulating crosstalk between these signaling pathways in cancer [25]. We then examined whether ZIF-8 induces crosstalk of apoptotic pathways through CAS-6. Immuno-fluorescent staining was performed to demonstrate that higher levels of CAS-6 were present in SCC7 cells (Figure 6A–C) following treatment with 100 μg/mL ZIF-8 compared to DPSCs (Figure 6D–G). To further confirm whether ZIF-8-induced activation of apoptotic pathways in SCC7 is mediated by death receptor-related signaling cascades, we next examined the levels of Caspase-10, a crucial initiator caspase molecule in death receptor pathways [26]. Immunofluorescent staining was performed to demonstrate that higher levels of CAS-10 were present in SCC7 cells (Figure 7A–C) following treatment with 100 μg/mL ZIF-8 compared to DPSCs (Figure 7D–G). Collectively, our findings revealed that ZIF-8 treatment can induce apoptosis in SCC7, but not in OMSCs, through programmed cell death pathways related to death receptor signaling.

## 4. Discussion

The present study provides valuable insights into the selective cytotoxicity of ZIF-8, particularly its differential effects on oral cancer (SCC7) and OMSC (DPSC and PDLSC) cells. The precise role of MSCs in tumor biology—whether they have tumor-suppressive effects or promote tumor growth—has not reached consensus within the scientific community [15]. Nevertheless, it is well established that OMSCs are integral components of the oral environment, maintaining tissue homeostasis, stability, and self-renewal properties under physiological conditions [27]. Therefore, an important property of chemotherapeutic agents would be to have an inhibitory effect on malignant cells while minimizing damage to OMSCs and other surrounding stromal tissues. Our findings suggest that ZIF-8 demonstrates this characteristic, exerting significant inhibition of OSCC viability while largely sparing OMSC populations, highlighting its potential as a targeted therapy for oral cancer treatment.

The selective cytotoxicity of ZIF-8 against SCC7, most clearly depicted at a concentration of 100 μg/mL ZIF-8, is consistent with our prior findings [22]. Through nuclear staining, SCC7 exhibited a drastic cytotoxic effect at 100 μg/mL ZIF-8, compared to lower concentrations and the vehicle control. In contrast, OMSC populations, including DPSCs and PDLSCs, exhibited negligible cytotoxic effects at the same concentration. Similar effects of ZIF-8 on stem cells have been demonstrated in the context of bone scaffolding and nerve repair. ZIF-8 has shown good biocompatibility with MSCs and other bone-related cells [28], supporting adhesion, proliferation, and even osteoinductive potential [29]. In addition, ZIF-8 was shown to enhance axonal outgrowth in DPSC-derived neuro-like cells and confer resistance to apoptosis in transplanted DPSCs under injury conditions. It is believed that ZIF-8 activates the mitogen-activated protein kinase (MAPK) signaling pathway in DPSCs, promoting neural differentiation and angiogenesis [16]. The differential response between cancerous and stromal cells is crucial, as it may reduce the adverse effects typically associated with traditional chemotherapies that indiscriminately target both cancerous and normal cells [30]. This dichotomy further suggests that ZIF-8 could potentially be developed as a treatment with reduced systemic toxicity.

To further characterize the effects of ZIF-8 on both types of cells, LIVE/DEAD assays were performed at the optimal concentration of 100 μg/mL ZIF-8. While the proportion of nonviable DPSCs remained relatively stable, the proportion of nonviable SCC7 cells was significantly higher, indicating a potent anticancer effect of ZIF-8. This selective cytotoxicity suggests that ZIF-8 may serve as a promising candidate for targeted cancer therapy, potentially reducing the need for aggressive treatments that affect healthy surrounding tissues. In a study by Johari et al., the cytotoxicity of ZIF-8 was tested on two eukaryotic cell lines, including human embryonic kidney (HEK293) and human colon cancer (SW480) cells. They found that the toxicity of ZIF-8 nanoparticles to both cell lines increased noticeably as the exposure concentration rose from 0 to 500 μg/mL. They also observed that ZIF-8 nanoparticles caused a greater reduction in the viability of human colon cancer cells compared with human embryonic kidney cells. Specifically, the increase in cell death upon ZIF-8 exposure was associated with elevated reactive oxygen species (ROS) production and activation of apoptotic pathways [31]. Our findings also raised the important mechanistic question of how ZIF-8 induces cancer cell death.

To elucidate the mechanism, an immunohistochemical analysis of apoptosis-related proteins (BAX, CAS-3, CAS-6, and CAS-10) was conducted. The cellular uptake kinetics of ZIF-8 nanoparticles are generally characterized by rapid, time-dependent initial internalization over the first few hours [32]. Studies have shown that ZIF-8 may be internalized via a clathrin-mediated pathway [33], but further research is needed to more specifically identify the mechanisms by which ZIF-8 induces cell apoptosis. Specifically, the BAX protein inhibits the activity of anti-apoptotic Bcl-2 proteins, leading to activation of the mitochondrial apoptosis pathway [34]. Caspases are a group of endo-proteases that play key roles in the regulatory networks governing inflammation and apoptosis [35]. Both BAX and caspases were upregulated in SCC7 cells exposed to 100 μg/mL ZIF-8. These findings indicate activation of the intrinsic mitochondrial apoptotic pathway in SCC7 cells following ZIF-8 exposure. In contrast, these apoptotic markers were less pronounced in DPSCs, further supporting the conclusion that ZIF-8 selectively induces apoptosis in SCC7 cells while sparing DPSCs. While the current study utilizes immunocytochemistry and RNA-seq to provide a robust overview of ZIF-8′s selective toxicity, we acknowledge that these results represent a snapshot of cellular response at 72 h. Incorporating additional complementary techniques, such as Western blot or quantitative PCR, would further strengthen and validate the present findings.

The acidic pH of a tumor acts as a trigger for ZIF-8, causing it to degrade more readily in the tumor microenvironment than in healthy tissues. This selective release not only improves targeting and reduces harm to normal cells but also results in the release of zinc ions, which stimulate ROS production in cancer cells [36,37]. It is plausible that ZIF-8-mediated zinc ion release may increase intracellular ROS, which could contribute to mitochondrial dysfunction and apoptotic signaling [36]; however, direct ROS measurements were not performed in this study and warrant future investigation. Moreover, ZIF-8 itself or its components, such as zinc, may further enhance its anticancer effects by serving as cofactors for enzymatic processes or by increasing oxidative stress [37]. In the present study, all assays were conducted at a 72 h time point, selected based on preliminary optimization experiments and prior work demonstrating robust ZIF-8–mediated cytotoxic and apoptotic effects at this time point. Earlier time points (e.g., 12–48 h) may provide additional insight into the temporal dynamics of early apoptotic signaling and upstream molecular events. Future studies incorporating time-course analyses will be important to further delineate the kinetics of ROS generation, caspase activation, and death receptor signaling pathways.

## 5. Conclusions

ZIF-8 serves not only as a drug delivery system but also holds significant promise as a targeted therapeutic agent for oral cancer, exhibiting a selective cytotoxic effect on cancerous SCC7 cells while sparing the viability of OMSC populations. Collectively, ZIF-8 at 100 μg/mL has an inhibitory effect on SCC7 but no significant effect on the viability or apoptosis of OMSCs. Further investigation using in vivo animal models will be essential to fully evaluate the translational potential of ZIF-8. In vivo studies would allow assessment of biodistribution, tumor-targeting efficiency, systemic toxicity, biodegradation kinetics, and potential effects on surrounding normal oral tissues within a physiologically relevant tumor microenvironment. Moreover, deeper mechanistic explorations, such as ROS generation, zinc ion release kinetics, receptor-mediated apoptotic signaling, and pathway-specific inhibition studies, would provide a more comprehensive understanding of the molecular events underlying ZIF-8–induced apoptosis. These studies will be critical for validating the safety, specificity, and therapeutic efficacy of ZIF-8 as a targeted delivery platform for oral cancer treatment.

## Data Availability

The original contributions presented in this study are included in the article. Further inquiries can be directed to the corresponding author.

## Abbreviations

The following abbreviations are used in this manuscript:

5-FU	5-fluorouracil
α-MEM	Alpha minimum essential medium
BAX	Bcl-2–associated X protein
BMI-1	B lymphoma Mo-MLV insertion region 1
CAF	Cancer-associated fibroblasts
CAS3	Caspase-3
CAS6	Caspase-6
CAS10	Caspase-10
DPSC	Dental pulp stem cell
EthD-1	Ethidium homodimer-1
FBS	Fetal bovine serum
FC	Fold change
GO	Gene ontology
GSEA	Gene set enrichment analysis
HNSCC	Head and neck squamous cell carcinoma
LIVE/DEAD	LIVE/DEAD viability/cytotoxicity assay
MAPK	Mitogen-activated protein kinase
MSC	Mesenchymal stem/stromal cell
OMSC	Orofacial mesenchymal stem/stromal cell
OSCC	Oral squamous cell carcinoma
OCT4	Octamer-binding transcription factor 4
PBS	Phosphate-buffered saline
PDLSC	Periodontal ligament stem cell
PFA	Paraformaldehyde
RNA-seq	RNA sequencing
ROI	Region of interest
